# Imputing missing distances in molecular phylogenetics

**DOI:** 10.7717/peerj.5321

**Published:** 2018-07-24

**Authors:** Xuhua Xia

**Affiliations:** Department of Biology, University of Ottawa, Ottawa, Ontario, Canada; Ottawa Institute of Systems Biology, University of Ottawa, Ottawa, Ontario, Canada

**Keywords:** Distance matrix, Imputing missing distance, Least-squares method, Phylogenetics

## Abstract

Missing data are frequently encountered in molecular phylogenetics, but there has been no accurate distance imputation method available for distance-based phylogenetic reconstruction. The general framework for distance imputation is to explore tree space and distance values to find an optimal combination of output tree and imputed distances. Here I develop a least-square method coupled with multivariate optimization to impute multiple missing distance in a distance matrix or from a set of aligned sequences with missing genes so that some sequences share no homologous sites (whose distances therefore need to be imputed). I show that phylogenetic trees can be inferred from distance matrices with about 10% of distances missing, and the accuracy of the resulting phylogenetic tree is almost as good as the tree from full information. The new method has the advantage over a recently published one in that it does not assume a molecular clock and is more accurate (comparable to maximum likelihood method based on simulated sequences). I have implemented the function in DAMBE software, which is freely available at http://dambe.bio.uottawa.ca.

## Introduction

Distance-based phylogenetic methods, especially those based a local or global optimization criterion (Desper & Gascuel, 2002; Desper & Gascuel, 2004; Saitou & Nei, 1987), are widely used in studies on molecular phylogenetics and evolution. The least-square method for phylogenetic reconstruction is generally consistent when the distance is estimated properly (Felsenstein, 2004; Gascuel & Steel, 2006; Nei & Kumar, 2000), and is quite robust against over- or under-estimated distances (Xia, 2006). The popularity of the distance-based methods arises not only from their speed and performance which allows them to build super-trees (Criscuolo et al., 2006; Criscuolo & Gascuel, 2008), but also from their applicability to non-sequence data (Wayne, Van Valkenburgh & O’Brien, 1991). In particular, distance-based methods represent the only category of methods that can construct a phylogeny based only on pairwise alignment (Thorne & Kishino, 1992), which may be valuable in situations when reliable multiple alignment is difficult to obtain with highly diverged taxa. Such a phylogenetic method based on pairwise alignment has been implemented in DAMBE (Xia, 2013; Xia, 2017) for nucleotide, codon and amino acid sequences.

There are cases where distance-based methods are the only option for building phylogenetic trees, such as those involving the new genome-based distances proposed in recent years. These include genome BLAST distances (Auch et al., 2006; Deng et al., 2006; Henz et al., 2005), breakpoint distances based on genome rearrangement (Gramm & Niedermeier, 2002; Herniou et al., 2001), distances based on the relative information between unaligned/unalignable sequences (Otu & Sayood, 2003), distances based on the sharing of oligopeptides (Gao & Qi, 2007), the composite vector distance (Xu & Hao, 2009), and composite distances incorporating several whole-genome similarity measures (Lin et al., 2009).

**Figure 1:**
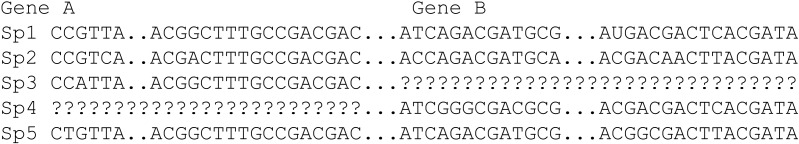
A sequence data set from concatenating Gene A and Gene B sequences. A distance cannot be computed between Sp3 and Sp4 because they share no homologous sites.

Distance-based methods may be the only way to build phylogenetic tree even with sequence data. For example, thousands of DNA transposons exist in Tasmanian devil (Gallus et al., 2015), but many have accumulated so many indels and substitutions that it is impossible to obtain a multiple alignment. The analysis is then limited to computing the distance between the consensus and each individual sequences (Gallus et al., 2015), without being able to have a phylogenetic tree. One can do pairwise alignment among most of the transposon sequences and compute their distances, but some transposon sequence pairs do not share homologous sites (Fig. 1, where a distance between Sp3 and Sp4 cannot be computed) and therefore cannot have their distances computed. If these missing distances can be imputed from those computable ones, then we have a method (and the only one) to build a phylogeny from such sequences. The same scenario is found in bacteriophage where (1) many do not share homologous genes and (2) high sequence divergence precludes multiple sequence alignment (but pairwise alignment using dynamic programming is often possible).

Note that missing data in this manuscript does not refer to indels in aligned sequences. In the distance matrix context, missing data means that some distances in the distance matrix are missing. In the sequence context, missing data means lack of homology between sequences to compute evolutionary distances. For sequences where a reliable multiple alignment can be obtained, likelihood-based methods are expected to have better phylogenetic accuracy than distance-based method, with or without imputed distances.

## Methods

### The statistical rational

Suppose we have *N* species with *K* possible pairwise distances, where *K* = *N*(*N* − 1)∕2. Also suppose that *M* distances are missing and need to be imputed. The general framework for imputing missing distances is to find the *M* distances corresponding to the best tree based on certain criteria. There are two criteria used in choosing the best tree: the least-squares (LS) criterion (Beyer et al., 1974; Cavalli-Sforza & Edwards, 1967) and the minimum evolution (ME) criterion (Rzhetsky & Nei, 1992). I will show that only the LS criterion is appropriate for imputing missing distances.

I will first outline the general approach, point out problematic cases where unique solution cannot be found, and then develop an efficient computational method which partially resembles the expectation–maximization (EM) algorithm. However, this approach is easily trapped in a local optimum and a downhill simplex method in multidimensions (Press et al., 1992, pp. 408–412) was implemented for imputing multiple missing distances. I illustrate the method by applying it to real data.

**Figure 2:**
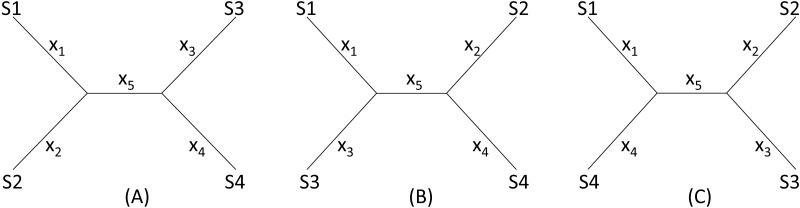
Topologies for illustrating distance imputation, with three possible unrooted topologies designated (A), (B) and (C) for four species labelled S1–S4.

I will start with a simple illustrative example. Suppose we have four species (S1 to S4 in Fig. 2) with *D*_12_ = 2, *D*_14_ = 5, *D*_23_ = 3, *D*_24_ = 5, *D*_34_ = 4 but with *D*_13_ missing. One may take a wrong approach by thinking that, in this particular case, we have five unknowns and five equations and can solve for *D*_13_ exactly. For example, given a topology in Fig. 2A, we can write the expected *D*_ij_ values, i.e., *E*(*D*_ij_), as:

(1) \begin{eqnarray*}\begin{array}{@{}l@{}} \displaystyle E({D}_{12})={x}_{1}+{x}_{2}\\ \displaystyle E({D}_{14})={x}_{1}+{x}_{5}+{x}_{4}\\ \displaystyle E({D}_{23})={x}_{2}+{x}_{5}+{x}_{3}\\ \displaystyle E({D}_{24})={x}_{2}+{x}_{5}+{x}_{4}\\ \displaystyle E({D}_{34})={x}_{3}+{x}_{4} \end{array}\end{eqnarray*}

These *E*(*D*_ij_) values are termed patristic distances in phylogenetics. If we replace *E*(*D*_ij_) by the observed *D*_ij_ values, we can indeed solve the simultaneous equations in Eq. (1), which give the solution as

(2) \begin{eqnarray*}\begin{array}{@{}l@{}} \displaystyle {x}_{1}= \frac{{D}_{12}}{2} + \frac{{D}_{14}}{2} - \frac{{D}_{24}}{2} \\ \displaystyle {x}_{2}= \frac{{D}_{12}}{2} + \frac{{D}_{24}}{2} - \frac{{D}_{14}}{2} \\ \displaystyle {x}_{3}= \frac{{D}_{23}}{2} + \frac{{D}_{34}}{2} - \frac{{D}_{24}}{2} \\ \displaystyle {x}_{4}= \frac{{D}_{34}}{2} + \frac{{D}_{24}}{2} - \frac{{D}_{23}}{2} \\ \displaystyle {x}_{5}= \frac{{D}_{14}}{2} + \frac{{D}_{23}}{2} - \frac{{D}_{12}}{2} - \frac{{D}_{34}}{2} \end{array}\end{eqnarray*}

The missing *D*_13_ given the tree in Fig. 2A, designated as *D*_13.A_, can therefore be inferred, as:

(3) \begin{eqnarray*}{D}_{13.A}={x}_{1}+{x}_{5}+{x}_{3}={D}_{14}+{D}_{23}-{D}_{24}.\end{eqnarray*}

Thus, given the five known *D*_ij_ values above, I obtain *x*_1_ = *x*_2_ = *x*_3_ = *x*_5_ = 1, *x*_4_ = 3, *D*_13.A_ = 3. The tree length (*TL*), defined as *TL* = ∑*x*_*i*_, is 7 for the tree in Fig. 2A, i.e., *TL*_A_ = 7. *TL* is used in the ME criterion for choosing the best tree. The best tree is one with the shortest *TL*.

One might think of applying the same approach to the other two trees in Figs. 2B, 2C to obtain *D*_13.B_ and *D*_13.C_ as well as *TL*_B_ and *TL*_C_, and choose as the best *D*_13_ and the best tree by using either the LS criterion or the ME criterion (Rzhetsky & Nei, 1992; Rzhetsky & Nei, 1994), i.e., the tree with the shortest *TL*.

This approach has two problems. First, the approach fails with the tree in Fig. 2B where the missing distance, *D*_13_, involves two sister species. One can still write down five simultaneous equations, but will find no solutions for *x*_*i*_, given the *D*_ij_ values above, because the determinant of the coefficient matrix is 0. For the tree in Fig. 2C, the solution will have *x*_5_ =  − 1. A negative branch length is biologically meaningless and defeats the ME criterion for choosing the best tree and the associated estimate of *D*_13_. Second, in most practical cases where missing distances are imputed, there are more equations than unknowns, e.g., when we have five or more species with one missing distance.

The LS approach aims to find the missing distances and the topology that minimizes the residual sum of squared deviation (RSS):

(4) \begin{eqnarray*}RSS=\sum \frac{{ \left[ {D}_{ij}-E \left( {D}_{ij} \right) \right] }^{2}}{D_{ij}^{m}} \end{eqnarray*}

where *D*_ij_ is the distance that can be computed from species i and j (i.e., not missing), *E*(*D*_ij_) is specified in Eq. (1) for the tree in Fig. 2A, *m* is a constant typically with a value of 0 (ordinary least-squares, OLS), 1 (Beyer et al., 1974), or 2 (Cavalli-Sforza & Edwards, 1967). In the illustration below, I will take the OLS approach with *m* = 0. It has been shown before that OLS actually exhibits less topological bias than alternatives with *m* equal to 1 or 2 (Xia, 2006).

Given the three tree topology, the results from the LS estimation are summarized in Table 1. Note that, for the tree in Fig. 2B, there are multiple sets of solutions of *x*_*i*_ that can achieve the same minimum RSS of 1.

**Table 1:** Estimation results from minimizing RSS, with Trees A, B, and C as in Fig. 2, and with the constraint of no negative branch lengths.

Site	Tree A	Tree B	Tree C
*x*_1_	1	0	1
*x*_2_	1	1.5	1.5
*x*_3_	1	0	1
*x*_4_	3	3.5	3.5
*x*_5_	1	1	0
D_13_	3	0	2
TL	7	6	7
RSS	0	1	1

We see a conflict between the LS criterion and the ME criterion in choosing the best tree and the best estimate of *D*_13_. The ME criterion would have chosen Tree B with *TL*_B_ = 6 and *D*_13_ = 0 because *TL*_B_ is the smallest of the three TL values. In contrast, the LS criterion would have chosen Tree A with RSS = 0 and *D*_13_ = 3. There is no strong statistical rationale for the ME criterion, which is based on the assumption that substitutions are typically rare in evolution, so a tree with few substitutions is more likely than a tree requiring many substitutions. However, this criterion is logically inappropriate for imputing distances because it favors the distance that is the smallest. Phylogeneticists sometimes think that the ME criterion would be appropriate if the branch lengths are not allowed to take negative values (Desper & Gascuel, 2002; Desper & Gascuel, 2004; Felsenstein, 1997). The illustrative example in Table 1 shows that the ME criterion is problematic even when I do not allow negative branch lengths. In contrast, the LS-criterion (or the best-fit criterion) is well-established.

An earlier version of DAMBE implemented the LS approach above by using an iterative approach similar to the EM (expectation–maximization) algorithm as follows. For a given distance matrix with a missing distance *D*_ij_, I simply fill in the missing *D*_ij_ by the smallest sum of *D*_ik_ and *D*_jk_. For example, if *D*_25_ is missing, but I have a {*D*_23_, *D*_35_} pair and a {*D*_27_, *D*_57_} pair with (*D*_23_+ *D*_35_) < (*D*_27_ + *D*_57_), then (*D*_23_ + *D*_35_) is used as the initial *D*_25_. According to triangular inequality, *D*_25_ ≤ (*D*_23_ + *D*_35_). These initial *D*_ij_ guesstimates are designated as *D*_ij,m0_ where the subscript “m0” indicates missing distances at step 0. I now build a tree from the distance matrix that minimizes RSS in Eq. (4). From the resulting tree I obtain the patristic distances *E*(*D*_ij_) from the tree and replace *D*_ij.m0_ by the corresponding *E*(*D*_ij_) values which are now designated as *D*_ij.m1_. I now build a tree again, obtain the corresponding *E*(*D*_ij_) to replace *D*_ij.m1_, so now I have *D*_ij.m2_. I repeat this process until RSS does not decrease any further. This process can quickly arrive at a local minimum. Unfortunately, different topologies have different minimums, and this approach is too often locked in a local minimum with a tree that does not achieve a global minimum RSS.

I have implemented the LS criterion with a downhill simplex method in multidimensions (Press et al., 1992, pp. 408–412) when multiple distances are missing. With a single missing distance, the Brent’s method (Press et al., 1992, pp. 402-408) is used. The optimization is run multiple times, with different initial values for the points in the simplex to increase the chance of finding the global RSS associated with the missing distances and the tree. While the simplex method is slow, it is good for proof-of-principle studies. The next version of DAMBE will replace the simplex method by the faster Powell’s method (Press et al., 1992, pp. 412–419).

**Figure 3:**
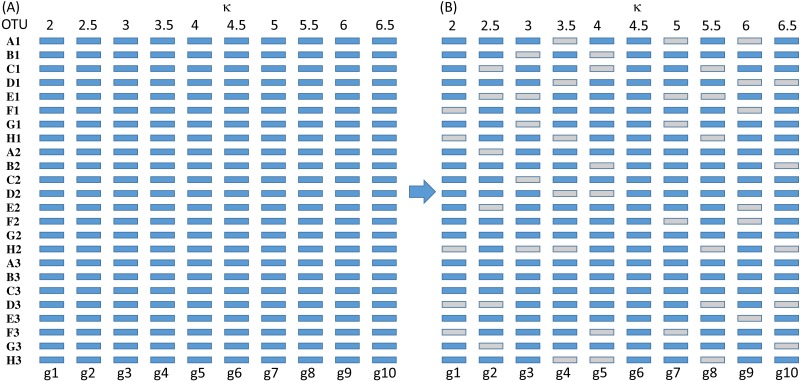
Sequence configuration for each set of sequences before deletion (A) and after (B).

### Comparison against the maximum likelihood method with simulated sequences

The pruning algorithm used for computing the likelihood can handle missing data, which was numerically illustrated in detail (Xia, 2014). While this method is intended in cases where a reliable multiple sequence alignment is difficult to obtain, i.e., when the maximum likelihood (*ML*) method is inapplicable, it is still of interest to gauge the performance of the distance imputation and phylogenetic reconstruction against the *ML* method.

The simulated sequences consist of 24 OTUs and 10 genes evolving in the HKY85 model (Hasegawa, Kishino & Yano, 1985) but with different transition bias (different *κ* values) varying from 2 to 6.5 (Fig. 3). The simulation was performed with INDELible 1.03 (Fletcher & Yang, 2009) with a symmetrical topology. I attach the supplemental control.txt file that specifies the specifics of the simulation including substitution models, nucleotide frequencies, indel rate and distribution, and phylogenetic tree with branch lengths. Each simulated set of sequences is aligned with MUSCLE (Edgar, 2004) with the default option (which is the slowest but most accurate). I have also used MAFFT (Katoh, Asimenos & Toh, 2009) with the LINSI option that generates the most accurate alignment (‘–localpair’ and ‘–maxiterate = 1,000’). Alignment from MAFFT generally contains more indels than MUSCLE but the phylogenetic results from the sets of alignments are almost identical.

Each of the 10 genes were simulated independently generating 1,000 sets of sequences with each set containing 24 OTUs (and 24 simulated sequences). They are then concatenated into 1,000 sets of sequences, with each sequence being a concatenation of 10 genes in the configuration shown in Fig. 3. The first 100 sets of sequences without gene deletion is designated Group0 (Fig. 3A). The next 100 sets of sequences with one gene randomly deleted (out of the 10 concatenated genes in Fig. 3) is designated Group1, and so on. The 100 sets with N genes randomly deleted is designated GroupN, where N varies from 0 to 9. The simulated data in 10 files named Group0.fas, Group1.fas, …, Group9.fas are in supplemental file Group0_9.fas.zip.

Maximum likelihood phylogenetic reconstruction was performed with PhyML (Guindon & Gascuel, 2003). The tree improvement option ‘-s’ was set to ‘BEST’ (best of NNI and SPR search). The ‘-o’ option was set to ‘tlr’ which optimizes the topology, the branch lengths and rate parameters. The distance imputation and distance-based phylogenetic analysis was done in DAMBE by choosing simultaneously estimated distance (Tamura, Nei & Kumar, 2004; Xia, 2009; Xia & Yang, 2011) and FastME as the tree building algorithm. To replicate results from this method in DAMBE, click ‘File—Open file with multiple data sets” to open a GroupX.fas file. Specify 24 as the number of sequences per set, and then choose “Distance-based phylogenetics” to perform phylogenetics analysis with DAMBE defaults on all data sets in the file. PhyML can be run in DAMBE in a similar way. The resulting trees are then compared against the “true tree” used in simulation. I used the bipartition-based Robinson-Foulds’ method for tree comparison. Recovering a true tree also recovers all bipartitions in the true tree. A reconstructed tree that differs from the true tree also implies that some bipartitions in the true tree are not recovered. The Robinson-Foulds method of tree comparison can be accessed in DAMBE by clicking “Phylogenetics—Robinson-Foulds dist between trees”.

## Results

### Distance matrix input

**Figure 4:**
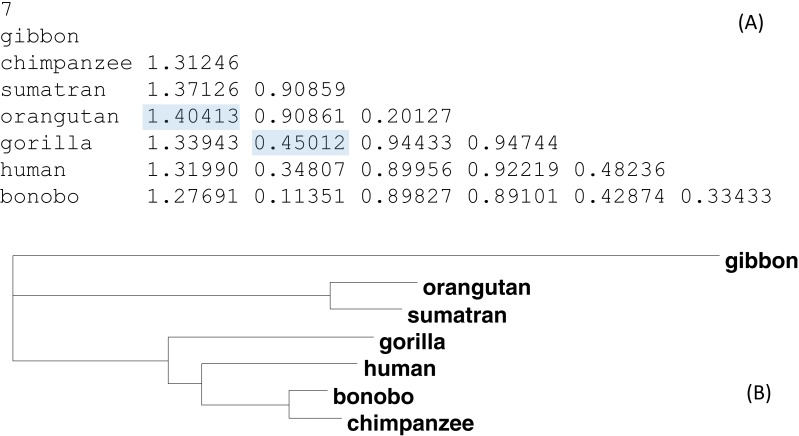
An example data set for imputing missing distances. (A) A real distance matrix computed from aligned sequences, but we pretend that the two shaded distances are missing. (B) A phylogenetic tree from the distance matrix.

Figure 4 shows an illustrative example with seven OTUs (operational taxonomic units). The distance matrix in Fig. 4A is computed from aligned sequence data used before (Xia & Yang, 2011). Figure 4B is the phylogenetic tree built from this distance matrix. Suppose *D*_gibbon,orangutan_ and *D*_gorrila.chimpazee_ are missing (shaded in Fig. 4A) and need to be imputed. The method above yields *D*_gibbon,orangutan_ = 1.3776 and *D*_gorrila.chimpazee_ = 0.4600, which are close to the observed values (Fig. 4A). The final tree built from the distance matrix with the two missing distances is identical to Fig. 4B except for a negligible difference in branch lengths. Note that I have two distances missing out of a total of 21 possible pairwise distances, which suggests that phylogenetic reconstruction is possible with nearly 10% of distances missing.

### Sequence input

I used a set of mitochondrial COI and CytB sequences from 10 Hawaiian katydid species in the genus *Banza* together with four outgroup species (Table 2) to test the performance of distance imputation by the method detailed above. Two sequence files are provided as supplemental file: (1) COI_CytB_aln.fas file that contains both COI and CytB sequences for each specimen, and (2) COI_CytB_aln_withMissing.fas that excluded sequences whose accession numbers are in strikethrough font in Table 2. There are 24 OTUs (Table 2), with 18 OTUs having COI sequences and 19 OTUs having CytB sequences. Out a total of 276 possible pairwise distances, 30 OTU pairs do not share homologous sites and need to have their distances imputed. This is a more dramatic example than before with more than 10% of the distances missing.

**Table 2:** Katydid species, GenBank accession, and sequence length (L) of COI and CytB genes. The suffixes A, B and C indicate different specimens from the same species.

Species	ACCN^a^	L_COI_	L_CytB_	Distribution
*Banza nihoa_A*	DQ649491 , DQ649515	1,233	729	Nihoa
*B. nihoa_B*	DQ649492, DQ649516	1,255	729	Nihoa
*B. kauaiensis_A*	DQ649483, DQ649507	1,255	729	Kauai
*B. kauaiensis_B*	DQ649484, DQ649508	1,255	729	Kauai
*B. unica_A*	DQ649501, DQ649525	1,255	729	Oahu
*B. unica_B*	DQ649502, DQ649526	1,117	729	Oahu
*B. parvula_A*	DQ649497, DQ649521	1,255	748	Oahu
*B. parvula_B*	DQ649498, DQ649522	1,254	748	Oahu
*B. molokaiensis_A*	DQ649487, DQ649511	1,255	695	Molokai
*B. molokaiensis_B*	DQ649488, DQ649512	1,255	659	Molokai
*B. deplanata_A*	DQ649481, DQ649505	1,255	686	Lanai
*B. deplanata_B*	DQ649482, DQ649506	1,255	686	Lanai
*B. brunnea_A*	DQ649479, DQ649503	1,255	748	West Maui
*B. brunnea_B*	DQ649480, DQ649504	1,255	747	West Maui
*B. mauiensis_A*	DQ649485, DQ649509	1,255	744	West Maui
*B. mauiensis_B*	DQ649486, DQ649510	1,255	748	West Maui
*B. pilimauiensis_A*	DQ649499, DQ649523	1,255	729	East Maui
*B. pilimauiensis_B*	DQ649500, DQ649524	1,255	729	East Maui
*B. nitida_A*	DQ649493, DQ649517	1,255	747	Hawaii
*B. nitida_B*	DQ649495, DQ649519	1,222	705	Hawaii
*B. nitida_C*	DQ649494, DQ649518	1,255	690	Hawaii
*R. lineosa*	NC_033991	1,534	1,137	East Asia
*R. dubia*	NC_009876	1,537	1,137	East Asia
*Neoconocephalus sp*	DQ649489, DQ649513	1,117	748	America

**Notes.**

a Two accession numbers are for partial COI and CytB sequences, respectively. One accession number is for the mitochondrial genomic sequence from which full-length COI and CytB sequences are extracted. Those with a strike-out font are “missing”, i.e., removed from aligned sequences for testing the effect of missing data, so some OTUs, e.g., *B. nihoa_B* and *B. kauaiensis_B,* do not share homologous sites and need to have their distance imputed.

**Figure 5:**
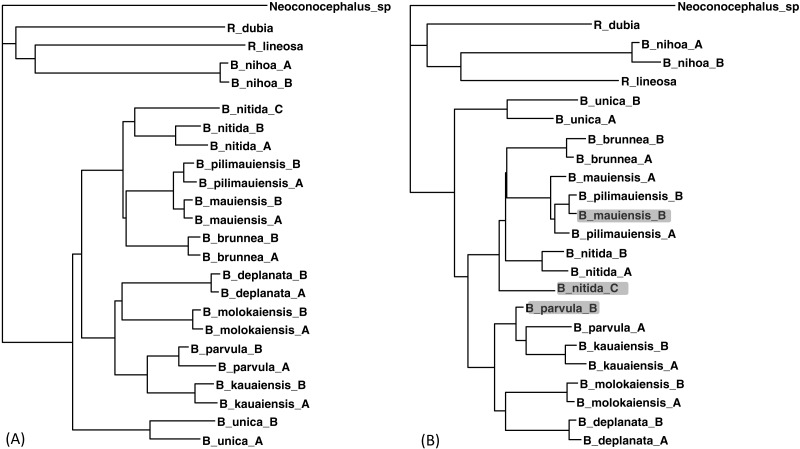
Phylogenetic performance from imputed distances. Comparison between a tree with all distances known (A) and another with 30 distances missing (B), reconstructed with the FastME method implemented in DAMBE. The neighbor-joining tree, also implemented in DAMBE, is the same. Three differences in OTU placement were highlighted in (B).

The two sequences were read and analyzed in DAMBE with the simultaneously estimated distances based on the TN93 model (MLCompositeTN93). The missing distances are then imputed. The final output tree, based on the distance matrix with the imputed distances, is reconstructed with either the FastME method (Desper & Gascuel, 2002; Desper & Gascuel, 2004) or the neighbor-joining method (Saitou & Nei, 1987). The tree with 30 distances missing (Fig. 5B) is generally consistent with the tree with the full data set (Fig. 5A) except three minor misplacements of OTUs (shaded in Fig. 5B). Giving the missing sequences indicated in Table 2, I can only get a tree of 18 OTUs with the COI data, and a tree of 19 OTUs with CytB data. By imputing missing distances, I can obtain a tree with 24 OTUs that is almost as good as the tree with the full data set.

In addition to the purging of sequences shown in Table 2, I have also purged sequences in different ways. The result that distance imputation and phylogenetic reconstruction can be done satisfactorily with about 10% of distances missing is generally repeatable.

### Contrasting performance against maximum likelihood method for aligned sequences

**Figure 6:**
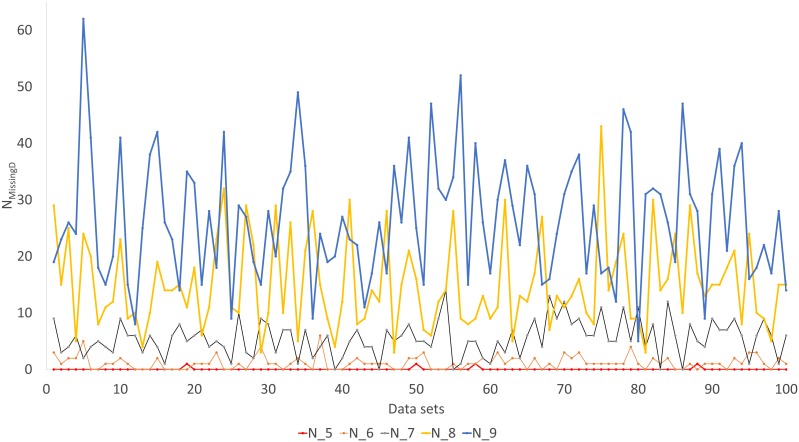
Number of missing distances in data sets (N_MissingD_) with different intensity of gene deletion (N5 to N9 standing for five to nine genes randomly deleted from each sequence containing 10 concatenated genes).

Simulated sequence data are grouped into Group0 to Group9. Each group contain 10 sets of aligned sequences, with no gene deletions in sequence sets in Group0, but with progressively more gene deletions from Group1 to Group9, leading to progressively more missing distances (Fig. 6). Sequence sets in Group0 to Group4 data do not have missing distance to impute, although deletion of gene sequences occur in sequences from Group1 to Group4. This is because sequences share at least one gene with either other for distance computation. Sequence sets in Group0 to Group5 always recover the true tree with either PhyML or the method of distance-imputation plus FastME reconstruction.

**Figure 7:**
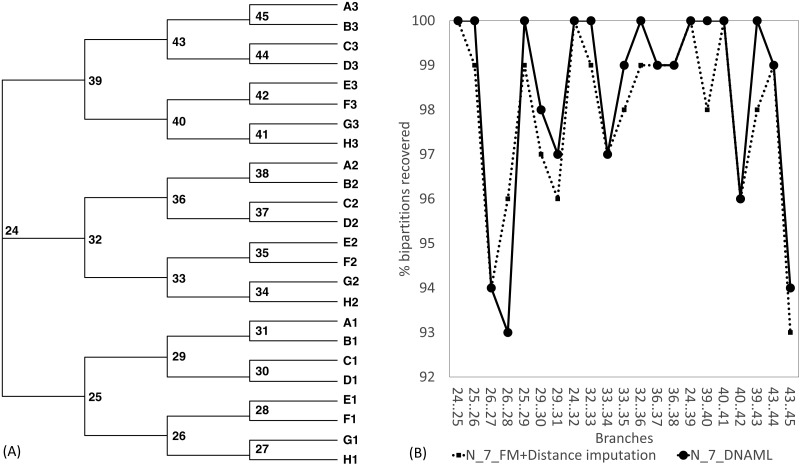
Illustration of the Robinson-Foulds method of comparing phylogenetic accuracy. (A) A balanced tree with node labels, with 21 possible bipartitions, used in sequence simulation. A bipartition is created by cutting one internal branch, e.g., cutting the branch between nodes 24 and 25 (designated as 24..25) creates a bipartition with OTUs A1 to H1 in one partition and all other OTUs in the other. (B) % of bipartitions (created by cutting the branch specified in *X*-axis) recovered from simulated sequences, based on (1) distance-based method FastME in conjunction with distance imputation (FM+Distance imputation) and (2) the maximum likelihood method (DNAML), for the 100 data sets when seven of the 10 concatenated genes are randomly deleted (N_7_MF +Distance imputation versus N_7_DNAML).

The Robinson-Foulds method used here to assess phylogenetic accuracy is based on tree bipartitions. A bipartition is generated when a branch is broken to separate a tree into two subtrees. A tree with 24 OTUs have 21 bipartitions. Cutting the branch between nodes 24 and 25 (designated as 24..25) creates a bipartition with OTUs A1 to H1 in one partition and all other OTUs in the other. A tree with 24 OTUs as in Fig. 7A has 21 bipartitions. If a reconstructed tree has the same topology as the true tree, then all 21 bipartitions will be identical between the two trees. Thus, the percentage of bipartitions in a true tree (which is used to simulate the sequences) recovered from simulated sequences is a proxy of phylogeny accuracy. Figure 7B shows one special comparison between the distance-based method with imputed distances and the maximum likelihood method, with seven of the 10 concatenated genes randomly deleted from each sequence. The two approaches recovered a high and comparable percentage (93-100%) bipartitions in the true tree. The corresponding lines for data sets with fewer gene deletions are expected to be closer to 100%, and those for data sets with more gene deletions are expected to have lower percentages.

**Figure 8:**
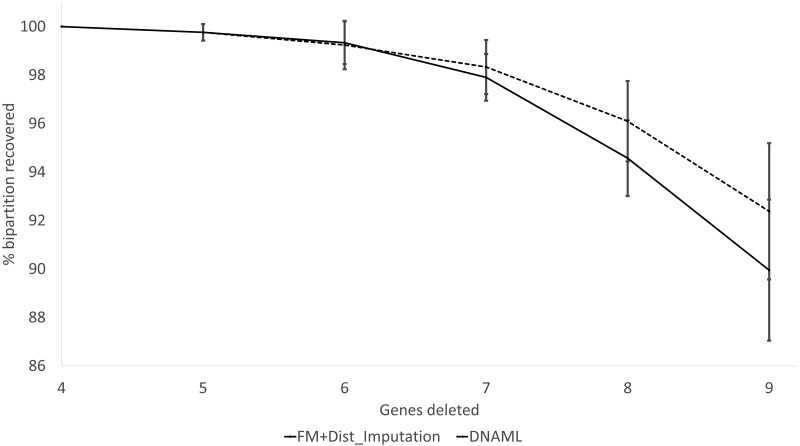
Percentage of bipartitions in the true tree recovered by the reconstructed trees, contrasting between (FM + Distance imputation) and DNAML. The percentage decreases with increasing number of genes randomly deleted in the data sets leading to increasing number of missing distances. One standard deviation of the percentage is shown for each point.

These expected patterns are empirically substantiated in Fig. 8 for Group5 to Group9. The percentage of recovered bipartitions decreases with increasing number of gene deletion (which is associated with an increasing number of missing distances). The distance-based method (FastME) based on imputed distances on average is worse than the likelihood-based method (represented by DNAML in Fig. 8), especially with more genes randomly deleted.

The results from simulated data are consistent with the previous deletion involving the COI and CytB genes. Given the variation in the number of missing distances in Fig. 6, the distance imputation and phylogenetic reconstruction is comparable to that of the maximum likelihood (Fig. 7 and Fig. 8), albeit slightly worse. The pattern suggests that the distance-based method with imputed distances should be used in cases involving up to about 10% of missing distances.

## Discussion

Imputing 30 missing distances does highlight the speed limitation of the simplex method of optimization, which is known to be the slowest (but simplest to implement) in multivariable optimization (Press et al., 1992, pp. 408–412). It takes almost 1.5 min to complete the distance imputation and phylogenetic reconstruction on my desktop PC with a i7-4770 processor clocked at 3.4 Ghz. If the number of missing distances is reduced to 15, then the computation is instantaneous.

There are cases where missing distances can only be determined approximately. For example, if our OTUs include avian species and mammalian species and if distances between mammalian species are missing, then there will be distances that have a narrow range of optimal values instead of a single optimal value. Any distance value within that range will lead to the same minimum RSS. The only way to eliminate this problem is not to have sister species with a missing distance.

There is another program, Lasso, for building phylogenetic trees from a distance matrix with missing values (Kettleborough et al., 2015). I found Lasso to be inaccurate. First, Lasso does not recover the tree in Fig. 4B. Second, the tree for the katydid species, when constructed with Lasso, differs in numerous ways from the tree in Fig. 5A. Lasso assumes a molecular clock, probably because it uses a UPGMA-type of phylogenetic reconstruction. I did not investigate whether Lasso’s performance is limited by the assumption of molecular clock assumption or in distance imputation.

While missing data can be accommodated by the likelihood method with the pruning algorithm (Felsenstein, 1973; Felsenstein, 1981, pp. 253–255; 2004), they can inflate branch lengths and introduce phylogenetic bias (Darriba, Weiss & Stamatakis, 2016; Xia, 2014). Some popular likelihood-based phylogenetic methods, e.g., PhyML (Guindon & Gascuel, 2003), optionally use distance-based methods to build the initial phylogenetic tree, which is then modified in various ways and evaluated in the likelihood framework to find the maximum likelihood tree. Distance-based methods are much faster than other phylogenetic methods such as maximum likelihood, Bayesian inference and maximum parsimony, and consequently are useful in constructing supertrees.

## Conclusion

Distance imputation and phylogenetic reconstruction can be done with about 10% of distances missing, and the phylogenetic result is almost as good as that with full information. The method in the paper has an advantage over a previous method (Kettleborough et al., 2015) that assumes a rooted tree and a molecular clock for building a tree and for inferring missing distances. This assumption is not needed and is too restrictive in practice.

## Software Availability

DAMBE is available free at http://dambe.bio.uottawa.ca. It can take two types of distance data. The first is the distance matrix data in PHYLIP format, but with missing distances represented by ‘.’ (a period without quotation marks). One can access the function of distance imputation by clicking ‘File—Open other molecular data—Distance matrix file with missing values’, and open a distance matrix file. DAMBE will output the imputed distance together with the best tree.

The second input type sequence data, either aligned or unaligned. DAMBE reads and converts almost all currently used sequence formats. For aligned data, DAMBE will compute the distances between sequences sharing homologous sites, impute distances between sequence pairs that do not share homologous sites, and output the imputed distances and the associated optimal tree. For unaligned sequences, DAMBE will align homologous sequences, compute their pairwise distances, impute distances from those sharing no homologous sites, and output the imputed distances and the optimal tree. This function is accessed by clicking ‘Phlogenetics—Sequence aligned’ or ‘Phylogenetics—Phylogenetics by pairwise alignment’.

##  Supplemental Information

10.7717/peerj.5321/supp-1 Supplemental Information 1 Aligned and concatenated mitochondrial COI and CytB sequences

10.7717/peerj.5321/supp-2 Supplemental Information 2 Aligned and concatenated mitochondrial COI and CytB sequences with missing data Aligned and concatenated mitochondrial COI and CytB sequences with six COI and five CytB sequences missing.

10.7717/peerj.5321/supp-3 Supplemental Information 3 Control file specifying details of sequence simulation Control file specifying details of sequence simulation, used with INDELible 1.0.3.

10.7717/peerj.5321/supp-4 Supplemental Information 4 Simulated sequences for replicating Figs. 6 and 7 Simulated data organized in 10 files named Group0.fas, Group1.fas, ..., Group9.fas, with no gene deletion in Group0.fas, but progressing to more gene deletion from Group1.fas to Group9.fas. Can be used to replicate Figs. 6 and 7

## Additional Information and Declarations

### Data Availability

The following information was supplied regarding data availability: The raw data are provided in the Supplemental Files.
